# Genome-wide association study identifies new susceptible loci of IgA nephropathy in Koreans

**DOI:** 10.1186/s12920-019-0568-6

**Published:** 2019-08-19

**Authors:** Kyung Hwan Jeong, Jin Sug Kim, Yu Ho Lee, Yang Gyun Kim, Ju-Young Moon, Su Kang Kim, Sun Woo Kang, Tae Hee Kim, Sang Ho Lee, Yeong Hoon Kim, Representing the KNOW-CKD Study Group

**Affiliations:** 1 Division of Nephrology, Department of Internal Medicine, Kyung Hee University School of Medicine, 26, Kyungheedae-ro, Dongdaemun-gu, Seoul, South Korea; 2 Department of Biomedical Laboratory Science, Catholic Kwandong University, Gangneung, 25601 Republic of Korea; 3 Division of Nephrology, Department of Internal Medicine, Inje University College of Medicine, 633-165, Gaegum-dong, Busanjin-gu, Busan, Republic of Korea

**Keywords:** IgA nephropathy, Susceptibility loci, Genome-wide association study

## Abstract

**Background:**

Immunoglobulin A nephropathy (IgAN) is the most common primary glomerulonephritis worldwide. Recent evidence suggests that genetic factors are related to the pathogenesis of IgAN. We conducted a genome-wide association study (GWAS) to identify novel genetic susceptibility loci for IgAN in a Korean population.

**Methods:**

We enrolled 188 biopsy-confirmed IgAN cases and 455 healthy controls for the discovery stage and explored associations between IgAN and single nucleotide polymorphisms (SNPs) using a customized DNA chip. The significant SNPs from the discovery samples were then selected for replication in an independent cohort with 310 biopsy-confirmed IgAN cases and 438 healthy controls.

**Results:**

In the first stage, two SNPs (rs10172700 in *LOC105373592* and rs2296136 in *ANKRD16*) were selected for further association analysis in the next stage. In the replication cohort, rs2296136 in *ANKRD16* was significantly associated with IgAN (odds ratio [OR] = 1.40, 95% confidence interval [CI] 0.99–1.98, *p* = 0.05 in log-additive model, OR = 1.55, 95% CI = 1.06–−2.27, *p* = 0.02 in dominant model, and OR = 0.70, 95% CI = 0.17–−2.84, *p* = 0.62 in recessive model). rs2296136 in *ANKRD16* also showed a significant association with IgAN in the entire study population combining GWAS and replication study (*p* = 0.0045 in log-additive model, *p* = 0.0027 in dominant model, and *p* = 0.76 in recessive model).

**Conclusions:**

The SNPs identified in the present study could be good candidate markers for predicting IgAN in Koreans, although further experimental validation is needed.

## Background

Immunoglobulin A nephropathy (IgAN) is the most common primary glomerulonephritis worldwide [1]. Its clinical features vary, and it has been recognized as an important cause of kidney failure [1, 2]. The prevalence of IgAN varies substantially according to geographic region [3]. Individuals of Asian descent are more likely to be affected than individuals from other ethnic backgrounds [2]. Familial clustering of IgAN has also been recognized throughout the world [4]. Moreover, some studies have demonstrated immunologic defects and urinary abnormalities in asymptomatic family members of patients with IgAN [4, 5]. Taken together, these findings suggest that genetic factors strongly influence the pathogenesis of IgAN.

In the last two decades, candidate gene association studies and linkage studies seeking candidate genes for IgAN [6, 7] have reported several candidate genes involved in glycosylation, immune regulation, and cytokine pathway. However, these studies have some sample size limitations and methodological problems [7]. Recently, genome-wide association studies (GWASs) have recognized several susceptibility loci for IgAN [8–11]. GWASs enable the identification of common alleles in complex disease. In contrast to prior studies, GWASs have been shown to identify susceptibility variants even in the setting of significant locus heterogeneity [2]. However, there are some limitations inherent in GWASs. First, GWASs detect only common disease-causing variants that have relatively small effect size. Second, most of the loci are noncoding, and many are located far from the discovered genes. Third, GWASs are not always replicated across studies or populations. Lastly, the previous GWAS DNA chips were fixed and offered less coverage of single nucleotide polymorphisms (SNPs) in exon and promoter regions.

The aim of this study was to identify novel genetic susceptibility loci for IgAN in a Korean population using a customized DNA chip, containing mostly exon and promoter region. We conducted a two-stage GWAS of biopsy-confirmed IgAN with 188 cases and 455 healthy controls in the discovery phase and with independent cohort of 310 cases and 438 healthy controls in the validation phase of the two significant SNPs. To overcome the restrictions inherent in small sample size, we selected biopsy-confirmed IgAN patients from multiple centers.

## Method

### Study design and subjects

We conducted a two-stage GWAS of IgAN in a Korean population. The first stage (discovery cohort) consisted of a GWAS, and the second stage (validation cohort) was a replication analysis of the top SNP signals that were identified during discovery. Samples with a call rate < 97% or gender mismatch were removed for sample quality control in both the first and second stages. The first stage included 188 patients with biopsy-confirmed IgAN from three kidney centers (Kyung Hee University Medical Center, Seoul, Korea; Kyung Hee University Hospital at Gangdong, Seoul, Korea; and Inje University Busan Paik Hospital, Korea) and 455 healthy controls from the general health check-up program. The second stage included 310 patients with biopsy-confirmed IgAN from the KoreaN Cohort Study for Outcomes in Patients With Chronic Kidney Disease (KNOW-CKD) cohort and 438 healthy controls from the general health check-up program. The KNOW-CKD study is a multicenter, prospective cohort study of adults with CKD in Korea; the study design has been described previously [12]. The healthy controls enrolled in both stages were recruited from the general health check-up program using patients with 1) normal renal function (estimated glomerular filtration rate > 90 mL/min/1.73 m^2^, and 2) no evidence of kidney injuries in the urine analysis, and 3) no structural problems in the kidney. We calculated the statistical sample power of discovery set using a genetic power calculator (http://osse.bii.a-star.edu.sg/calculation2.php).

All study procedures complied with the ethical guidelines of the 1975 Declaration of Helsinki, as revised in 2000. The study protocol was approved by the Institutional Review Board of all centers, and the approval number was 2012–01-130 obtained from Kyung Hee University Hospital at Gangdong. Written informed consent was obtained from all participants.

### Design of the customized DNA chip

**Fig. 1:**
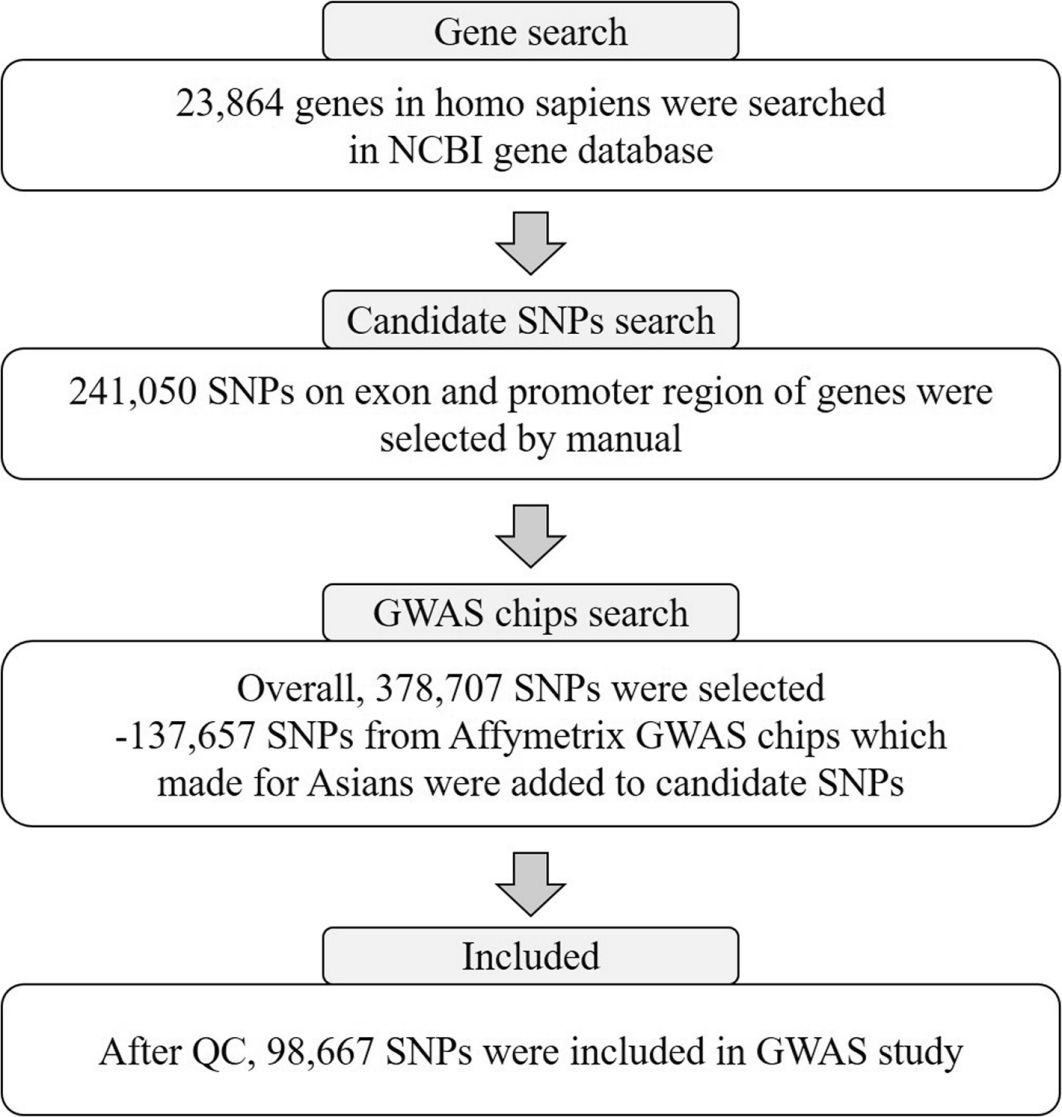
Workflow of genetic selection and creation of the customized DNA chip. SNP, single nucleotide polymorphism; GWAS, genome-wide association study; DNA, deoxyribonucleic acid

As shown in Fig. 1, we first selected 23,864 *Homo sapiens* genes from the NCBI gene database (https://www.ncbi.nlm.nih.gov/gene) and manually searched for all known SNPs in all selected genes using the dbSNP database (https://www.ncbi.nlm.nih.gov/snp/). Finally, 241,050 candidate SNPs that were previously reported to be associated with human diseases such as glomerulonephritis, malignancies, autoimmune disorders, and psychiatric disorders were included in this study. We considered regions (exon and promoter regions) for candidate SNPs to surmount the limitation of previous GWAS DNA chips. Candidate SNPs selected according to the following criteria: (1) SNPs located in exon and promoter regions in each gene; (2) SNPs studied in previous GWASs or case and control studies with various diseases; (3) SNPs reported in Asians; (4) SNPs with > 10% minor allele frequency (MAF) in Asian; (5) > 0.1heterozygosity. We then, added 137,657 SNPs from Affymetrix (Affymetrix, CA, USA) GWAS chips which provide high genetic coverage in East Asian Populations. We finally designed a customized chip using the Axiom™ Genome-Wide Human Assay.

### DNA isolation, genotyping, and quality controls

Genomic DNA was extracted from peripheral blood samples collected in tubes coated with EDTA using a commercially available Roche DNA extraction Kit (Roche, IN, USA). We used the Customer Axiom Exome Array by Affymatrix (Affymatrix, CA, USA) in order to genotype selected SNPs. The experimental process was carried out by Theragen, Suwon, Korea. The following exclusion criteria were applied for SNP quality control: a genotype call rate < 97% and a Hardy–Weinberg equilibrium *p*-value < 1 × 10^− 4^ in the controls. Overall, 98,667 SNPs remained after quality control.

### Replication analysis

In the second stage, the two significant SNPs identified from the first stage were genotyped. The criterion for candidate SNP selection was the association of a SNP with a p-value ≤5 × 10^− 5^ in our GWAS. Due to small sample size, there was no genetic association with a p-value less than 1 × 10^− 8^ that was a best-powered definition for the assessed number of SNPs. Genotyping of new samples from the independent cohort (310 cases and 438 controls) for validation was conducted by direct sequencing after genomic DNA was amplified using specific primers for each gene.

### Statistical analysis

In the GWAS analysis, association testing was done with PLINK using logistic regression in order to search candidate SNPs for IgAN in a Korean population (http://pngu.mgh.harvard.edu/~purcell/plink/). The quantile-quantile (Q-Q) and Manhattan plots were calculated using the statistical analysis program R (http://www.r-project.org/). In the replication phase, SNPstats (http://bioinfo.iconcologia.net/index.php) and SPSS 23.0 (SPSS Inc., Chicago, IL, USA) were used to calculate odds ratios (OR), 95% confidence intervals (CI), and *p*-value. Genetic models [dominant (major homozygous versus. Heterozygous + minor homozygous), recessive (major homozygous + heterozygous versus. Minor homozygous, and log-additive (major homozygous versus. Heterozygous versus. Minor homozygous) models] were applied.

## Results

### GWAS identifies two IgAN-susceptible SNPs in a Korean population

**Fig. 2:**
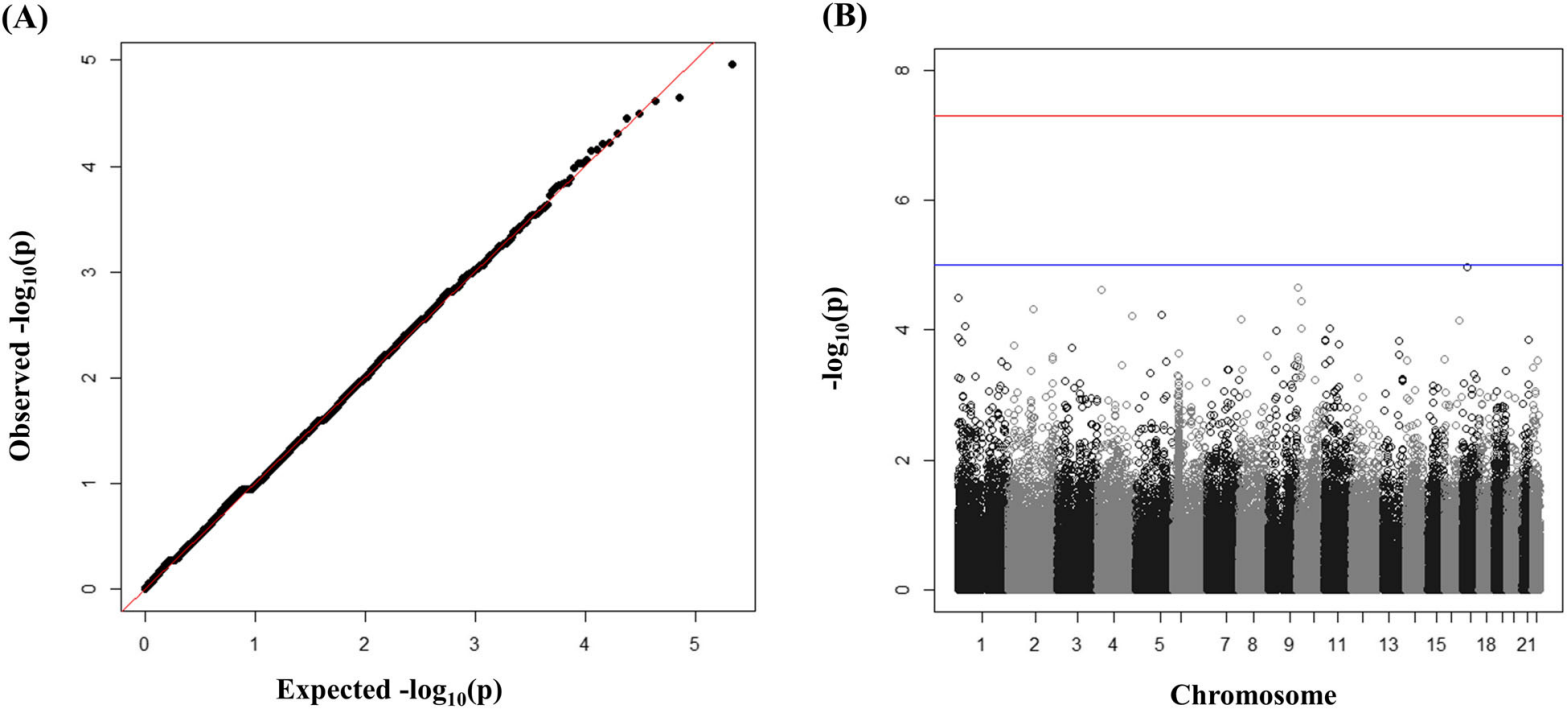
**a**, Quantile-quantile (Q-Q) plot of *p*-values for test statistics (Cochran-Armitage trend tests) in the GWAS. **b**, Manhattan plot showing the -log_10_*P* values of 98,667 SNPs in the GWAS for 188 IgAN patients and 455 healthy controls. GWAS, genome-wide association study; SNP, single nucleotide polymorphism; IgAN, Immunoglobulin A nephropathy

In the first stage, a GWAS analysis was performed with 188 IgAN cases and 455 healthy controls using 98,667 SNPs. The clinical characteristics of the cases and controls are summarized in Table 1. A Q-Q plot of observed versus expected *p*-values revealed significant associations between IgAN and certain SNPs (Fig. 2A). As shown in Fig. 2B, there was a significant signal of association with chromosome 6p. Because of the small sample number, there were no significant (*p* < 1 × 10^− 8^) gene associations with IgAN. For replication analysis, we excluded SNPs with MAF <  0.05, and selected only one of the most significant SNPs in the same gene. Two SNPs with suggestive evidence for association at *p* ≤ 5 × 10^− 5^ were selected for further association analysis (Table 2) in the second stage. The statistical sample power calculated using a genetic power calculator was 79.8% for rs10172700 and 75.1% for rs2296136.

**Table 1:** Clinical characteristics of IgAN patients and controls

	Discovery cohort		Validation cohort	
	IgAN (*n* = 188)	Control (*n* = 455)	IgAN (*n* = 310)	Control (*n* = 438)
Age (yrs)	36.1 ± 13.6	54.9 ± 16.3	44.1 ± 13.6	40.0 ± 5.1
Sex (n, M:F)	83: 105	210: 245	162: 148	255: 183
Pathologic stage (HS lee classification)	2.24 ± 1.02	ND	ND	ND
Creatinine (mg/dl)	1.00 ± 0.33	ND	1.49 ± 1.47	ND
24 h urine protein (mg/day)	1728.91 ± 2017.08	ND	1161.01 ± 1546.28	ND

*IgAN* Immunoglobulin A nephropathy, *ND* non-determined

**Table 2:** Summary of GWAS results and associations with IgAN in two selected SNPs

Gene symbol	SNP		Type	Control	IgAN	Model	OR (95% CI)	*p*-value
location/				n (%)	n (%)			
		Allele	A	563 (62.0)	181 (50.0)		1	
LOC105373592			G	339 (38.0)	181 (50.0)		1.66 (1.30–2.12)	5.00E-05
Intron	rs10172700		A/A	184 (40.8)	50 (27.6)	Log-additive	1.59 (1.26–2.02)	0.0001
		Genotype	A/G	195 (43.2)	81 (44.8)	Dominant	1.81 (1.24–2.63)	0.0017
			G/G	72 (16.0)	50 (27.6)	Recessive	2.01 (1.33–3.03)	0.0011
		Allele	G	850 (93.0)	311 (86.0)		1	
ANKRD16			C	60 (7.0)	51 (14.0)		2.32 (1.57–3.45)	1.92E-05
Exon (missense)	rs2296136		G/G	396 (87.0)	133 (73.5)	Log-additive	2.39 (1.59–3.59)	< 0.0001
		Genotype	G/C	58 (12.8)	45 (24.9)	Dominant	2.42 (1.58–3.72)	0.0001
			C/C	1 (0.2)	3 (1.7)	Recessive	7.65 (0.79–74.05)	0.053

*GWAS* genome-wide association study, *IgAN* Immunoglobulin A nephropathy, *SNPs* single nucleotide polymorphisms, *CHR* chromosome

### Replication study validates association of two SNPs with IgAN

To validate the association between the newly identified susceptible loci and IgAN in a Korean population, we conducted a replication study with the two SNPs identified from the first stage in an independent sample of 310 IgAN cases and 438 healthy controls (Table 1). Of the two selected SNPs from the prior stage, one SNP showed significant associations with IgAN: rs2296136 in *ANKRD16* (odds ratio [OR] = 1.40, 95% confidence interval [CI] 0.99–1.98, *p* = 0.05 in log-additive model, OR = 1.55, 95% CI = 1.06–2.27, *p* = 0.02 in dominant model, and OR = 0.70, 95% CI = 0.17–2.84, *p* = 0.62 in recessive model). We also analyzed the association between IgAN and the two selected SNPs in the entire study population (GWAS + replication study), and observed a significant association between IgAN and rs 2,296,136 in *ANKRD16* (*p* = 0.0045 in log-additive model, *p* = 0.0027 in dominant model, and *p* = 0.76 in recessive model) (Table 3).

**Table 3:** Association results of the two validated SNPs from GWAS in the replication cohort

Gene symbol location/	SNP	Genotype	Control	IgAN	Model	OR (95% CI)	p-value	Combined p-value (GWAS + replication)
			n (%)	n (%)				
LOC105373592	rs10172700	A/A	187 (42.7)	109 (35.2)	Log-additive	1.15 (0.94–1.41)	0.18	0.16
Intron		A/G	177 (40.4)	150 (48.4)	Dominant	1.37 (1.02–1.86)	0.03	0.04
		G/G	74 (16.9)	51 (16.4)	Recessive	0.97 (0.66–1.43)	0.87	0.99
*ANKRD16* Exon (missense)	rs2296136	G/G	373 (85.2)	244 (78.7)	Log-additive	1.40 (0.99–1.98)	0.05	0.0045
		G/C	59 (13.5)	63 (20.3)	Dominant	1.55 (1.06–2.27)	0.02	0.0027
		C/C	6 (1.4)	3 (1.0)	Recessive	0.70 (0.17–2.84)	0.62	0.76

*IgAN* Immunoglobulin A nephropathy, *SNPs* single nucleotide polymorphisms, *GWAS* genome-wide association study

### Associations between previously reported GWAS loci

We also performed an association analysis of IgAN with previously reported susceptible loci. Table 4 shows susceptible SNPs previously associated with IgAN and their references [6, 10, 11, 13–16]. Although we failed to demonstrate an association between IgAN and certain previously reported SNPs (rs6677604 in *CFH*, rs2523946 in *HCG9*, rs1883414 in *HLA-DPB2*, rs660895 in *HLA-DRB1*, rs2187668 in *HLA-DQA1*, rs2856717 in *HLA-DQB1*, rs2412971 in *HORMAD2*, rs11574637 in *ITGAX*, rs3803800 in *TNFSF13*, rs4227 in *SOX15*, rs252394 in *MICD*, and rs12537 in *MTMR3*), we found a modest association (*p* <  0.05) with other SNPs associated with IgAN in the *HLA-DRB1* and *HLA-DQB* genes (Table 4).

**Table 4:** Evidence of association with IgAN in Koreans for previously reported loci

			Previous report			Current study	
Gene	CHR	SNP	p-value	Study population	Reference	SNP	p-value
*CFH*	1	rs6677604	3.00E-10	Chinese	Zhu L et al. [13]	rs6677604	0.105780494
*HCG9*	6	rs2523946	2.00E-11	Han Chinese	Yu XQ et al. [11]	rs2523946	0.64876
*HLA-DPB2*	6	rs1883414	2.00E-11	European	Kiryluk K [10]	rs1883414	0.09046
*HLA-DRB1*	6	rs660895	4.00E-20	Han Chinese	Yu XQ et al. [11]	rs660895	0.00185
*HLA-DQA1*	6	rs2187668	3.00E-13	European	Ferreira RC [14]	rs2187668	0.16412
*HLA-DQB1*	6	rs2856717	1.00E-15	Chinese	Wang W et al. [6]	rs2856717	0.0395
*HORMAD2*	22	rs2412971	5.00E-12	European	Kiryluk K [10]	rs2412971	0.34740
*ITGAX*	16	rs11574637	8.00E-13	European	Kiryluk K [10]	rs11574637	1
*TNFSF13*	17	rs3803800	9.00E-11	Chinese	Yang C et al. [15]	rs3803800	0.52299
*SOX15*	17	rs4227	4.00E-10	Han Chinese	Yu XQ et al. [11]	rs4227	0.13883
*MICD*	6	rs2523946	5.00E-11	Han Chinese	Li M et al. [16]	rs252394	0.69067
*MTMR3*	22	rs12537	1.00E-11	Han Chinese	Yu XQ et al. [11]	rs12537	0.38984

*IgAN* Immunoglobulin A nephropathy, *CHR* chromosome, *SNPs* single nucleotide polymorphisms

## Discussion

Here, we present the results of a two-stage GWAS involving 492 biopsy-confirmed IgAN cases and 893 healthy controls. Despite the small sample size, our study is valuable in that we aimed to overcome the limitations of earlier studies by using a customized DNA chip, which is predominantly composed of exon and promoter regions and contained not only well-established but also unknown SNPs. In addition, we selected patients with biopsy-confirmed IgAN to overcome the small sample size. With this approach, we identified new susceptible loci of IgAN in the Korean population. The primary contribution from our study are: 1) we designed a customized DNA chip containing 98,667 SNPs; 2) we genotyped two candidate SNPs selected in the discovery stage using a validation cohort; and 3) we identified one susceptible SNP; rs2296136 in *ANKRD16*.

Despite remarkable progress since IgAN was first described by Berger et al. in 1968 [17], its pathogenesis has not yet been clearly defined. Inter-individual variation of disease course, differences in incidence among different ethnicities, and familial aggregation of the disease have suggested a genetic predisposition for IgAN [5]. In the last two decades, there have been many candidate-gene association studies and linkage analyses for IgAN [6, 7]. However, those studies were underpowered, and no specific causative mutations for IgAN have been identified. GWASs have recently emerged as an alternative approach, allowing for the identification of susceptibility loci that were previously unrecognized [18].

The first GWAS of IgAN was performed in subjects of European ancestry by Feehally et al. [8]. This study provided evidence for an association between IgAN and genes at HLA loci, across HLA-B, DRB1, DQA, and DQB. In several subsequent GWASs, nearly 20 risk variants for IgAN were identified (*CFHR1*, *CFHR3*, *HORMAD2*, *TNFSF13*, *DEFA*,*ITGAM-ITGAX*, *VAV3*, and *CARD9*, among others) [9–11]. Those loci are associated with the complement system, mucosal IgA production, and innate and acquired immunity [2]. However, these previously reported SNPs are GWASs that were fixed and had less coverage of SNPs in the exon and promoter regions.

Recently, several large-scale GWASs on the population of East Asia have been reported. Yu et al. [11] conducted a GWAS to identify susceptibility loci for IgAN in Han Chinese and showed that IgAN is associated with SNPs of near genes involved in innate immunity. This study group also performed the largest GWAS of IgAN in Han Chinese and identified new susceptibility loci (rs7634389 in *ST6GAL1*, rs2074038 in *ACCS*, and rs2033562 in *ODF1-KLF10*) [16]. The results of these previous studies have helped clinicians understand the pathogenesis of IgAN. However, considering the genetic differences and prevalence between East Asian countries, it is necessary to conduct GWAS of IgAN in the Korean population.

In the present study, we used the Axiom™ Genome-Wide Human Assay and found two SNPs with suggestive evidence for association with IgAN in the first stage (*p* ≤ 5 × 10^− 5^). Among these, rs2296136 in *ANKRD16* showed significant association with IgAN in the validation stage. *ANKRD16* is located at 10p15.1 and encodes the ankyrin repeat domain 16. Its function is unclear because only a few studies have investigated *ANKD16*. One study showed that *ANKRD16* is associated with subtype differences of breast cancer [19]. No study has reported an association between genetic variation in ANKRD16 and IgAN. We explored the effect of the variant on protein structure, function in missense SNPs and transcriptional activity in promoter SNPs. The probability of damage (probability > 0.8) for rs2296136 of *ANKRD16* was validated by polyphen2. Further functional studies are needed to elucidate whether ANKRD16 can affect IgAN.

Performing validation study of GWAS results is important for extending the effect estimation and providing acceptable statistical evidence [20, 21]. Although our study had small sample size in the discovery stage, we also validated our results using an independent samples consisting of 310 biopsy-confirmed IgAN cases and 438 healthy controls.

Our study has some potential limitations. First, this GWAS was conducted in a relatively small patient population. Because of the small sample size, statistically significant SNPs of *p* < 1 × 10^− 8^ were not found. However, we found SNPs that were presumed to be related to IgAN and proceeded to validation. In the existing GWAS study, the most significant SNPs were mostly rare SNPs, and these significant SNPs were not significant when tested in other groups. Genetic polymorphic markers based on DNA in precision medicine are very important. Race, sex, and other factors affect the significance of these SNPs for any given disease. Therefore, we cannot say that the SNPs found in this study are statistically highly significant, but the SNPs reported through these studies may help to find additional markers. As described, to compensate for the sample size, we selected patients with biopsy-confirmed IgAN. Second, we focused only on SNPs with a minor allele frequency greater than 0.05 and so might have missed rarer variations associated with IgAN. Third, the patients included in this study were predominantly Korean, so the results should be generalized with caution. Finally, we did not assay gene expression in vivo or examine functional effects according to genetic variants, relying instead on in silico functional detection software. To improve these weakness, we are planning a follow-up study using expression quantitative trait loci (eQTL) analyses. Interestingly, however, both promoter and missense functional assay programs showed that the rs2296136 variant of *ANKRD16* has important functional effects.

## Conclusions

This study is the first to identify a significant association between IgAN and a customized GWAS chip containing mostly exons and promoter regions. Several susceptible genetic loci suggest that these significant SNPs may be useful for investigating the pathogenesis of IgAN. The SNPs identified in the present study clarify the genetic architecture of IgAN and point to new pathogenic pathways.

## Data Availability

The datasets used and/or analyzed during the current study are available from the corresponding authors on reasonable request.

## Abbreviations

CI: Confidence interval
eQTL: Expression quantitative trait loci
GWAS: Genome-wide association study
IgAN: Immunoglobulin A nephropathy
LD: Linkage disequilibrium
MAF: Minor allele frequency
OR: Odds ratio
Q-Q: Quantile-quantile
SNPs: Single nucleotide polymorphisms
